# Variables and instruments for assessing mental health in competitive swimmers: A narrative review

**DOI:** 10.12688/f1000research.140504.2

**Published:** 2023-12-07

**Authors:** Alfonso Trinidad

**Affiliations:** 1 Aqualab Research Group, Campus Madrid, European University, Madrid, Community of Madrid, 28670, Spain; 2 Education and Humanities, Campus Madrid, European University, Madrid, Community of Madrid, 28670, Spain

**Keywords:** Natación, competición, deporte, bienestar, evaluación, supervisión, detección, prevención

## Abstract

**Background:**

Mental health issues are becoming increasingly prevalent among elite athletes, and during the Tokyo 2020 Olympic Games, this issue became a pressing concern. In particular, several athletes complained about their mental health in relation to the highly demanding demands of their sport. High-performance swimming exposes athletes to a variety of stressors due to the physical, technical and mental demands of the sport. The study has carried out a narrative review of the main variables related to mental health, their dimensions and evaluations in competitive swimmers.

**Methods:**

Clearly planned and ordered potential studies were identified using combined search methods. The search was carried out in different bibliographic databases (Dialnet, Web of Science and Scopus) between 1990 and 2023. Google Scholar was used to manually search the reference lists of the retrieved studies to identify potentially eligible studies that were not included in the electronic searches. The studies were examined from three different perspectives. Firstly, the methodology, sample, swimming categories and their relationship with mental health were analysed. Secondly, the variables related to mental health symptoms and disorders. Finally, the main mental health analysis tools and the conclusions of the studies.

**Results:**

The concept of mental health in competitive swimmers needs to be clarified in the scientific literature, as different variables have been analysed and multiple instruments have been used with small samples of swimmers, without any intervention and prevention protocol. In addition, the bodies and institutions involved should work in an interdisciplinary and collaborative manner, establishing specific programmes to ensure effective prevention and care.

**Conclusions:**

Studies are needed to fill this gap and it is necessary to work under the same consensus and in collaboration with specified technical teams. In order to monitor, evaluate and provide services through detection tests and support to swimmers in their training and competitions.

## Introduction

Mental health problems are increasingly present among elite athletes and it was during the Tokyo 2020 Olympics when it was an urgent call for this issue. In particular, several athletes complained about their mental health situation regarding the highly demanding inquiries about their sports modalities. Moreover, the World Health Organization (WHO) defines “global health” as “a state of complete physical, mental, and social well-being,”. This definition explicitly stresses that mental health is vital to our ability to think, feel and interact among us ( Jackson *et al.*, 2022; World Health Organization, 2018). However, far from this definition in terms of health, in high-competition swimming, the athletes are exposed to different stressors in high competition due to the physical, technical and mental demands ( Hooper *et al.*, 2022; Salazar *et al.*, 1997; Trinidad *et al.*, 2021). Likewise, it was also corroborated that psychological preparation and sports psychologists are essential factors within the technical teams with a view to the optimal performance of swimmers ( Rice *et al.*, 2016). It was not until 2017 that the International Olympic Committee (IOC) began to take care of the mental health of athletes, creating a specific working group (MHWG), to develop an evaluation and early recognition tool on the possible risks of suffering from mental health symptoms and disorders ( Gouttebarge *et al.*, 2021).

Since the 1990s, the scientific literature has emphasised mental health’s role among competitive swimmers. This factor is as important as physical health in optimising the athlete’s performance and well-being. However, this concept has been unnoticed by sports coaches and psychologists, due to the limited literature on swimming and the benefits of this sport for mental health ( Geamonond, 2018). The scarcity of research that has had competitive swimmers as an object of study has been a visible fact ( Jackson *et al.*, 2022; Rice *et al.*, 2016). Even in a recent review study ( Gouttebarge *et al.*, 2019), the absence of these athletes is evidenced by not characterizing them specifically ( Hughes & Leavey, 2012; Reardon & Factor, 2010). Similarly, high-quality systematic studies and intervention trials have been limited ( Rice *et al.*, 2016; Gouttebarge *et al.*, 2021), as well as descriptive research on the extent of mental health symptoms and disorders ( Gouttebarge *et al.*, 2019). This evidence set off alarms among swimmers, coaches and technical teams, as high rates of highly prevalent mental disorders begin to appear ( Rice *et al.*, 2016). This is contrary to the statement that swimming causes the sensation of well-being and relaxation, as well as control of anxiety, stress and fatigue ( Cordeiro *et al.*, 2017). Some factors include intense physical activity, overtraining, injuries and exhaustion ( Peluso & Andrade, 2005), proximity to sports retirement ( Gouttebarge *et al.*, 2016), a strict diet ( Sundgot-Borgen & Torstveit, 2004), physical and competitive stress, along with pressure from the public, the media, and the social media ( Bruner *et al.*, 2008; Fletcher & Wagstaff, 2009; Hanton *et al.*, 2005; Noblet & Gifford, 2002). Moreover, it should be added that athletes do not seek help for reasons such as a bad reputation, lack of understanding, the impact on performance and the perception that seeking help is a weakness ( Reardon & Factor, 2010; Gulliver *et al.*, 2012). This generates a high risk of mental illness that reduce sporting success ( Rice *et al.*, 2016).

Therefore, something must be fixed since the literature has warned us of the importance of psychological preparation and mental training ( Nideffer, 1985; Ryan, 1982). Moreover, putting up with the high training loads does not only depends on physical, technical and tactical preparation in order to achieve high performance ( Salazar *et al.*, 1997). Subsequently, the novelty of this study is to review the different works that examine the mental health of swimmers and the tools displayed to examine those characteristics of mental health in this population. To date, it seems that no previous review has examined the different tools and measures to portray competitive swimmers’ mental health situation. Therefore, this study aimed to carry out an analysis of the scientific tests taken with mental health in the discipline of swimming, as well as to examine the type of instrument used to evaluate and diagnose this situation in highly competitive swimmers.

## Methods

### Study design

A narrative review or literature review was carried out where the materials published in the literature (tests and instruments to evaluate and diagnose mental health in highly competitive swimmers) were described, in order to perform a textual, tabular or graphic synthesis of your results. and contributions ( Grant & Booth, 2009).

The start of the study was in February 2023 through combined search processes, in which potential studies, clearly planned and ordered, were identified. In orden to perform that, the following bibliographical databases were consulted: Dialnet, WOS (Web of Science) and Scopus with the following search terms included in Boolean search strategies: (mental*[tiab] AND (health [tiab] AND competitive [tiab] AND swimmers [tiab]) OR (swimming [tiab]) or in Spanish (mental*[tiab] AND (salud [tiab] AND competitivos [tiab] AND nadadores [tiab]) OR (natación [tiab]).

### Data collection

By using filter criteria of the respective databases, the search was limited to publication dates (from 1 January 1990 to 30 June 2023), species (humans), and English and Spanish languages. Moreover, Google Scholar was used for the search and a manual search of the reference lists of the retrieved studies was performed to identify potentially eligible studies that were not captured by the electronic searches.

To avoid internal biases in studies and between studies, the following criteria were adopted:

‐ Articles published from 1990 to 2023.
‐ Full-text articles written in English or Spanish languages (to reduce the bias of the revised publication) and peer-reviewed (to guarantee a minimum of manuscript quality and a minimum of reliability).
‐ Articles that analyzed competitive swimmers based on scientific methodology, with one or more measuring instruments in different variables.

Those that did not meet these criteria were excluded, as were books, book chapters, theses, thesis reviews, case study presentations, conference notes, and studies focused on non-competitive, amateur or triathlon swimmers. Also, it is important to note that research were only taken after extensively searching for existing research in that field.

### Data analysis

For collection and data organization, the tools offered by the different databases were used (text availability, article attributes, article type, publication date…). The data analysis was considered from three different points of view. Firstly, the methodology, sample, swimming categories (swimmers who competed in regional, university, national, international, masters championships and who had a minimum of 2 hours of training per week) were analysed and their relationship with mental health in competitive swimmers. Secondly, those variables (anxiety; motivation; self-esteem; goal orientation and goal setting; intensity and level of performance; importance of the test; coping; eating and depressive disorders; stress and recovery; exhaustion; tension, optimism and pessimism) that were related to mental health symptoms and disorders in competitive swimmers. Finally, the main analysis instruments for the mental health of competitive swimmers.

Table 1 shows the main research, as well as the methodology used, the sample and the swimming category of the analysed swimmers. Particularly, most studies followed a cross-sectional design (n = 8), followed by longitudinal (n = 4), comparative (n = 5) and a review (n = 1). Moreover, the sample was made up of 1174 swimmers. On the other hand, the swimming categories were: elite (n = 7), national/international (n = 3), master (n = 1), national (n = 1), university/international (n = 1), international (n = 1) and regional/national (n = 1). The period in which the studies were published was in a range of 27 years (from 1994 until June 2023).

**Table 1.** Methodologies, samples and swimming categories analysed and their relationship with mental health in competitive swimmers.

Author	Year	Methodology	Sample	Swimming category
Jones *et al.*	1994	Cross-sectional	*n* = 97 (Gender not specified)	Elite
Byrne & McLean	2002		*n* = 12 (Men = 4; Women = 8)	Elite
Abrahamsen *et al.*	2008		*n* = 190 (Men = 101; Women = 89)	Elite
Torstveit *et al.*	2008		*n* = 19 (Women)	Elite
Hammond *et al.*	2013		*n* = 50 (Men = 28; Women = 22)	University/International
Nixdorf *et al.*	2013		*n* = 1 (Not specified)	Elite
Hooper *et al.*	2022		*n* = 14 (Not specified)	Olympic
Mountjoy *et al.*	2022		*n* = 132 (Men = 67; Women = 65)	International
Hatzigeorgiadis & Chroni	2007	Longitudinal	*n = 39 (Men)*	International
Simões *et al.*	2012		*n = 9 (Men = 4; Women = 5)*	National/International
Didymus & Fletcher	2014		*n = 15 (Men = 8; Women = 7)*	National
Potdevin *et al.*	2015		*n = 490 (Men = 227; Women = 263)*	Master
Jones & Hanton	1996	Comparative	*n = 91 (Men = 45; Women = 46)*	National/International
Salazar *et al.*	1997		*n = 38 (Men = 14; Women = 24)*	National/International
Hanton & Jones	1999		*n = 8 (Men)*	Regional/National
Romberg *et al.*	2012		*n = 101 (Men = 55; Women = 46)*	Elite
Ning *et al.*	2022		*n = 47 (Women = 23)*	University/National
Jackson *et al.*	2022	Systematic review	*None*	None

## Results

**Figure 1.**
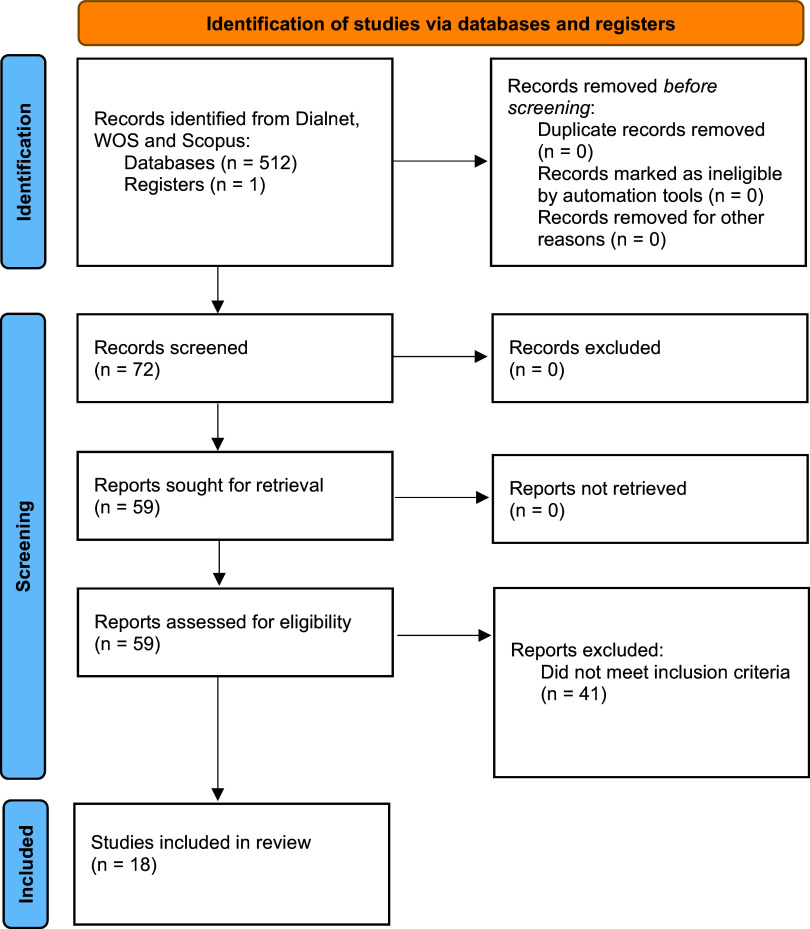
Identified articles flow chart.

Searches were made using titles, abstracts and keywords. The first comprehensive search yielded a total of 512 records. One additional article was identified by other means. After removing duplicates, 72 documents were identified, of which 41 were excluded as they were not directly related to the objective of the study. Finally, 59 articles were reviewed and, after applying the inclusion criteria, the 18 articles included in this review were selected ( Figure 1).

### Variables examined in previous works related to mental health in swimmers

Table 2 shows the main variables that examined the symptoms and disorders of mental health in competitive swimmers. The main studies focused on analyzing different variables such as: performance anxiety, as well as before and after competition; individual and group motivation; the self-esteem; orientation and goal setting; intensity and level of performance; the importance of the test; coping; eating and depressive disorders; stress and recovery; exhaustion, as well as tension, optimism and pessimism.

**Table 2.** Variables analysed about symptoms and mental health disorders in competitive swimmers.

Author	Variable
Jones *et al.* (1994)	Anxiety (performance, pre- and post-competition)
Jones & Hanton (1996)	Cognitive Anxiety pre-competition
	Cognitive Anxiety post-competition
	Goal Setting
Salazar *et al.* (1997)	Mood States
Hanton & Jones (1999)	Cognitive Anxiety and somatic post-competition
	Task Coping and sports performance
	Participation/purpose of the study
Byrne & McLean (2002)	Eating disorders
Hatzigeorgiadis & Chroni (2007)	Precompetitive anxiety, somatic anxiety y self-confidence
	Task Coping
Abrahamsen *et al.* (2008)	Task and ego orientation
	Perception of team climate, performance and specialty-performance
	Somatic anxiety, worry and concentration disorder
Torstveit *et al.* (2008)	Eating disorders
Romberg *et al.* (2012)	Psychosomatic index, life-esteem and self-esteem
Simões *et al.* (2012)	Goal settings
Hammond *et al.* (2013)	Depressive disorders
Nixdorf *et al.* (2013)	Depressive and associated factors
Didymus & Fletcher (2014)	Stress
Potdevin *et al.* (2015)	Health psychology, social and physical
Hooper *et al.* (2022)	Anxiety, exhaustion, psychological tension, and optimism/pessimism
	Coping with the cancellation of events
Mountjoy *et al.* (2022)	Depression
	Eating disorders
	Need for psychotherapy
	Experience of harassment
	Abuse in their sports environment
Ning *et al.* (2022)	Mindfulness level
	Flow state
	Anxiety, depression, training, and competition satisfaction

### Instruments used in the previous literature to analyse the symptoms and disorders experienced in competitive swimmers

Table 3 shows the main instruments used in the literature to analyse the possible symptoms and disorders associated with mental health in competitive swimmers. Particularly, the main variables examined in the studies analysed were: Anxiety (performance, pre- and post-competition) (n = 1), Mood (n = 1), Motivation (individual and groups) (n = 1), Self-esteem (n = 1), Goal orientation (n = 1), Goal setting (n = 1), Training intensity (n = 1), Performance level (n = 1), Test Importance (n = 1), Perceived efficacy coping (n = 1), Task Coping (n = 1), Coping with the events’ cancelation (n = 1), Eating disorders (n = 1), Depressive disorders (n = 1), Stress and recovery (n = 1), Exhaustion (n = 1), Psychological tension (n = 1), and Optimism and pessimism (n = 1). Regarding the main instruments utilized it stands out the use of the modified CSAI-2 (n = 1), the direction scale (n = 1), the mental readiness form (MRF-3) (n = 1), modified COPE (n = 1), the MCOPE (n = 1), the performance strategies test (TOPS) (n = 1), the semi-structured follow-up telehealth interviews (n = 1), the mental training program (MTP) (n = 1), the perception of success questionnaire (POSQ) (n = 1), the DSM-IV-TRir semi-structured questionnaire (n = 1), the BDI-II self-report instrument (n = 1), the Depression Scale CESD-R-10 (n = 1) and an online survey (n = 1).

**Table 3.** Main instruments for analyzing mental health symptoms and disorders in competitive swimmers.

Author	Instrument	Variable
Jones *et al.* (1994)	CSAI-2 modified	Precompetitive anxiety, somatic anxiety and self-confidence
Jones & Hanton (1996)	CSAI-2 modified	Cognitive and somatic anxiety prior to performance
	Direction scale	Post-performance somatic and cognitive anxiety
	Scale of expectations for the achievement of objectives	Goal settings
Salazar *et al.* (1997)	Profile of Mood States (POMS)	Mood states
Hanton & Jones (1999)	CSAI-2 modified	Post-performance somatic and cognitive anxiety
	Caree information questionnaire	Coping with the task and level of performance
	Social validation questionnaire	Participation/purpose of the study
Byrne & McLean (2002)	Composite International Diagnostic Interview (CIDI)	Eating disorders
	Subscales of Impulse to thinness, Bulimia, and Body dissatisfaction of the Eating Disorders Inventory II (EDI-II)	
	Bulimia Test-Revised (BULIT-R)	
	Restriction Subscale of the Three Dietary Factors Questionnaire (R)	
Hatzigeorgiadis & Chroni (2007)	Mental Preparation Form (MRF-3)	Precompetitive anxiety, somatic anxiety and self-confidence
	COPE and MCOPE modified	Coping with the task
	Performance Strategies Test (TOPS)	
Abrahamsen *et al.* (2008)	Success Perception Questionnaire (POSQ)	Ego and task orientation
	Questionnaire on Motivational Climate Perceived in Sport (PMCSQ)	Perception of team climate, performance and specialty/performance
	Sports Anxiety Scale (SAS-N)	Somatic anxiety, worry, and concentration disorders
Torstveit *et al.* (2008)	Questionnaire on menstruation, body weight, training, injuries and dietary history, use of oral contraceptives and pregnancy, physical activity patterns, nutritional habits, use of pathogenic weight control methods (PWCM) and self-declaration of eating disorders	Eating disorders
	EDI Body Dissatisfaction and Lean Impulse Subscales	
	Clinical interview and measurements of body composition, body fat percentage and fat-free mass (Dual-energy X-ray absorptiometry)	
	Eating Disorders Exam	
Romberg *et al.* (2012)	Questionnaire on asthma and breathing problems	Psychosomatic index, quality of life and self-esteem
Simões *et al.* (2012)	Mental training program (MTP)	Goal settings
Hammond *et al.* (2013)	Semi-structured interview based on the DSM-IVTR criteria	Depression
	Self-report instrument BDI-II	
Nixdorf *et al.* (2013)	Online survey	Depression and associated factors
Didymus & Fletcher (2014)	Journal of the Stress Assessment Record (SAL)	Stress
	Likert scale	
Potdevin *et al.* (2015)	Questionnaire SF 36	Psychological, social and physical health
	International Physical Activity Questionnaire (IPAQ)	
Hooper *et al.* (2022)	Different self-report scales	Anxiety, exhaustion, psychological tension, and optimism/pessimism
	Follow-up telehealth semi-structured interviews	Coping with the cancellation of events
Mountjoy *et al.* (2022)	Center for Epidemiological Studies Depression Scale revised (CESD-R-10)	Depression
	Brief Eating Disorders in Athletes Questionnaire (BEDA-Q)17	Eating Disorders
	Question “Have you ever wanted or needed support from a psychotherapist for personal or mental health problems?”	Need for psychotherapy
	Two questions: “Do you think harassment and/or abuse occurs in your sport?” “Have you ever witnessed or experienced yourself any forms of harassment or abuse in your sports environment?”	Harassment/abuse
Ning *et al.* (2022)	Five Facet Mindfulness Questionnaire	Level of mindfulness ability
	Short Flow State Scale	Challenge skill balance, action awareness, clear goal, clear feedback, focusing on the task at hand, sense of control, loss of self-awareness, time conversion, and enjoyable experience
	Competitive State Anxiety Inventory-2 (CSAI-2)	Anxiety, cognitive state anxiety, physical state anxiety, and state self-confidence
	Profile of Mood State	Tension, anger, fatigue, depression, energy, panic, and self
	Training and Competition Satisfaction Questionnaire	Training and competition satisfaction

### Causes of mental disorders in swimmers

Table 4 shows how the studies affirm that the high levels of physical activity that elite swimmers develop may cause high-stress levels, depression and anxiety, even after the competition cancellation, which psychological distress symptoms could follow. Other authors highlight that competitive swimming can lead to mental health problems, associated with other factors such as harassment and abuse, and the need to maintain a slim figure or a low body weight to compete. However, most of the studies agree that competitive swimmers should have a psychological support service and receive psychological preparation in this regard, which would help them as a tool for achieving goals and regulating anxiety in order to achieve a positive motivational climate that would allow them to reach a high degree of mental and physical well-being.

**Table 4.** Main contributions of the studies analysed.

Author	Main contributions of the studies
Jones *et al.* (1994)	The findings suggest that elite performers do not differ from non-elite performers on the intensity of pre-competition anxiety symptoms, but that they do have a more positive interpretation of these symptoms in terms of their consequences for performance. They also suggest that elite performers who do experience debilitative anxiety symptoms possess an effective cognitive strategy for maintaining confidence levels.
Jones & Hanton (1996)	The investigation has generated interesting findings that serve to understand further the relationship between goal attainment expectancies and debilitative and facilitative competitive anxiety. The findings also have practical implications for achieving appropriate preperformance states via goal-setting strategies. They emphasize, in particular, the importance of setting goals perceived as attainable and within the performer's control.
Salazar *et al.* (1997)	Psychological preparation is a fundamental element for optimal performance in elite athletes.
Hanton & Jones (1999)	The findings indicate that elite athletes experience and recall more demands associated primarily and directly with the sports organization than with competitive performance. Furthermore, this population appears more likely to mention similar competitive stressors but varied organizational stressors, probably because the former are inherent and endemic to elite sports, whereas the latter are essentially extraneous and widely distributed.
Byrne & McLean (2002)	The results suggest that athletes have a higher prevalence of eating disorders than non-athletes. However, it is not so much being an athlete that places an individual at increased risk for developing an eating disorder. Instead, athletes competing in sports that emphasise the importance of a thin body shape or a low body weight appear particularly vulnerable.
Hatzigeorgiadis & Chroni (2007)	The results suggest that facilitative perceptions of anxiety symptoms relate to more adaptive cognitive and behavioural outcomes. Coaches should therefore emphasize not only the regulation of anxiety intensity, but also the way swimmers perceive anxiety symptoms.
Abrahamsen *et al.* (2008)	The extant motivational climate affects performance anxiety, and coaches should consider this when working with elite national athletes.
Torstveit *et al.* (2008)	A higher prevalence of eating disorders was found among athletes competing in leanness sports compared with both athletes competing in non-leanness sports and controls. Therefore, we need to emphasize what kind of athletes we address when we talk about a higher prevalence of eating disorders in the sports environment. Using a risk criteria model including questions about menstrual dysfunction may be valuable in detecting Eating disorders in leanness sport athletes. However, it is sufficient to utilise self-report measures in non-leanness athletes.
Romberg *et al.* (2012)	Despite the increased asthma prevalence among the swimmers, they reported a high degree of mental and physical well-being, indicating that the sports environment per se compensated for eventual health drawbacks. Moreover, being active on the recreational level promotes a healthier lifestyle, an enhanced sense of well-being and fewer psychosomatic symptoms.
Simões *et al.* (2012)	Goal setting can be considered an overall context in which mental training can be integrated. It is more important for sport psychology to define the best psychological strategies in a single-subject perspective to cope with the stressful situations in sports.
Hammond *et al.* (2013)	The findings suggest that depression among elite athletes is higher than previously reported in the literature. Being ranked among the very elite athletes is related to an increase in susceptibility to depression, particularly in relation to a failed performance. Given these findings, it is important to consider the mental health of athletes and have appropriate support services in place.
Nixdorf *et al.* (2013)	The findings indicate that we can no longer deny the issue of depressive symptoms among elite athletes. Relevant factors such as chronic stress, coping strategies, and level of exhaustion and recovery may be practical starting points for establishing preventative programs and supportive interventions for depressed athletes.
Didymus & Fletcher (2014)	The results demonstrate the complexity of coping and support the notion that there may not be a ‘one size fits all’ answer to the question of which coping strategies are most effective in managing organizational stressors.
Potdevin *et al.* (2015)	The very high physical activity level in this competitive context did not result in significantly better levels on all parameters in comparison with their national counterparts (pain perception, social, emotional, and mental health).
Hooper *et al.* (2022)	Findings urge sports medicine clinicians to implement psychological screening protocols in elite athletes following the cancellation of a major sporting event to attend to symptoms of psychological distress and to direct appropriate psychological intervention.
Jackson *et al.* (2022)	Competitive swimming may be a promising conservative therapy for mental health management. However, further research is recommended to solidify these findings and establish the long-term effects of this intervention on mental health.
Mountjoy *et al.* (2022)	Targeted initiatives are required to address the burden of mental health issues and harassment and abuse in sport in the FINA aquatic disciplines.
Ning *et al.* (2022)	The MAIC mindfulness training program based on localization design has an obvious improvement effect on college “swimmers” mindfulness level, flow state, anxiety, depression, and training competition satisfaction, with a good continuity effect.

## Discussion

This study aimed to carry out an analysis of the scientific tests taken in mental health in the discipline of swimming, as well as to examine the type of instrument used to evaluate and diagnose this situation in highly competitive swimmers. In competitive swimming, mental health has been assessed from different perspectives and with great scientific heterogeneity. One of the possible causes could be associated with the fact that swimming is related to other areas of knowledge ( Navarro *et al.*, 1990), among which is psychology. The range of variables studied in this area has generated some uncertainty and overlooked certain factors that may have influenced swimmers’ vulnerability to mental health symptoms and disorders ( Bär & Markser, 2013). Likewise, some studies highlight that there are more than 640 different stressors ( Arnold & Fletcher, 2012) and that together with the sports withdrawal, they could develop a high probability of suffering from symptoms and mental health disorders ( Arnold & Fletcher, 2012; Wylleman & Reints, 2010). Therefore, it may be highlighted that mental health has not been analysed and evaluated in depth and rigour, according to the scientific literature. However, there are also controversies since other authors defend the idea that swimming positively affects general somatic health ( Oja *et al.*, 2015; Tanaka, 2009) and mental health ( Cordeiro *et al.*, 2017; Yfanti *et al.*, 2014). On the contrary, these opinions differ from those other researchers give when discussing mental health in high-level sports and its stressors and conditioning factors ( Henriksen *et al.*, 2020). For this reason, this scientific diversity has generated some uncertainty among researchers, not knowing what to focus their studies on when evaluating and diagnosing due to the dimensional amplitude of variables.

The reviewed scientific literature shows how the concept of mental health could be clearer between the studies, since most of them have analysed different variables separately ( Byrne & McLean, 2002; Didymus & Fletcher, 2014; Hammond *et al.*, 2013; Hooper *et al.*, 2022), to later refer to the term mental health in general. Likewise, most of these studies have focused mainly on variables such as: anxiety, coping, goal setting and depression ( Abrahamsen *et al.*, 2008; Nixdorf *et al.*, 2013; Salazar *et al.*, 1997; Simões *et al.*, 2012), neglecting other factors ( Arnold & Fletcher, 2012), which could also be analysed and give a more complete view in terms of mental health among swimmers. Therefore, it would be necessary to reach a consensus on mental health and work on these symptoms and disorders in an interconnected way with other factors (risk and recovery from injuries, sports performance, depression and anxiety) and relate them to other symptoms, injuries and physical illnesses ( Jones & Hanton, 1996). This could further clarify what mental health is and how it could be extrapolated to swimming, since most of the studies analysed in the literature focus on the study of a reduced number of variables or in the analysis of aquatic disciplines displayed by FINA ( Jackson *et al.*, 2022).

Likewise, the scientific literature seems to have not found a consensual categorization of the term mental health. Authors such as Rice *et al.* (2016) related mental health with: anger and aggression, anxiety, eating disorder and image, general prevalence studies, seeking help, sleep, stress and coping, substance use and well-being. In contrast, Gouttebarge *et al.* (2019) linked it to terms such as: distress, sleep, anxiety/depression, and alcohol abuse. Purcell *et al.* (2019) and Reardon *et al.* (2019) linked it with: high prevalence disorders (e.g., anxiety and mood symptoms) and more complex mental health disorders (e.g., bipolar and eating disorders). However, the International Olympic Committee (IOC) and its mental health working group (MHWG) have proposed a confidential mental health support service “Mentally Fit”. This service could help to clarify the term and establish different categories of analysis (pressure management, physical or psychological exhaustion, harmony between sport and life at home, management of life or career changes, stress management, communication improvement, how to deal with injuries, anxiety control, depression, eating disorders, bullying, harassment and abuse, and parenting). Also, this service could explain all the edges that make up mental health for future research, given the lack of consensus among researchers when categorising mental health. Furthermore, even the International Swimming Federation (FINA) does not have a program similar to that of the International Olympic Committee (IOC) and delegates, according to its general medical regulations, to the members of the Olympic movement. It is therefore questionable whether there is any concern on the part of this competent body for its swimmers in the area of mental health according to its current regulations.

It even reinforces the opinion of some researchers on the lack of concern on the part of federations and institutions ( Reardon & Factor, 2010), in the face of this situation in high-level swimmers. Therefore, it would be recommended that the International Swimming Federation (FINA) implement a health program that will help both coaches and athletes to improve their knowledge of mental health problems ( Mountjoy *et al.*, 2019), extending this to other aquatic disciplines under FINA conditions ( Jackson *et al.*, 2022).

Concerning the methodology used to detect mental health problems in swimmers, it has been shown that most cross-sectional, longitudinal and comparative studies do not correspond to high-quality research and intervention trials ( World Health Organization, 2018). This situation also corroborates the need for more consensus among researchers when it comes to intervening, preventing and establishing action protocols, to which is added the scarcity of scientific literature ( Trinidad *et al.*, 2021; World Health Organization, 2018), being necessary the expansion in this field through specific studies. Furthermore, it would be necessary to increase the analysis sample for future comparative studies ( Hainline & Reardon, 2019; Hammond *et al.*, 2013; Purcell *et al.*, 2019), since it was found that most research was at most 15 sample swimmers ( Henriksen *et al.*, 2020; Jackson *et al.*, 2022; Oja *et al.*, 2015; Tanaka, 2009).

Regarding the revised instruments for the measurement and diagnosis of mental health in swimmers, there is great heterogeneity since most of them have used between three and four measurement tools. First, for anxiety the following were mainly used: the modified CSAI-2 ( Ning *et al.*, 2022; Martens *et al.*, 1990), the direction scale, the mental readiness form (MRF-3) ( Krane, 1994) and different self-report scales ( Hooper *et al.*, 2022). Regarding coping, the following were used: the career information questionnaire, modified COPE ( Carver *et al.*, 1989) and MCOPE ( Crocker & Graham, 1995), the performance strategies test (TOPS) ( Hardy *et al.*, 2010) and the semi-structured follow-up telehealth interviews ( Ning *et al.*, 2022). In order to establish goals, the following were used: the scale of expectations of achieving objectives, the mental training program (MTP) and the perception of success questionnaire (POSQ) ( Roberts *et al.*, 1998). Finally, for depression, the following were used: the semi-structured interview based on the DSM-IV-TR criteria, the BDI-II self-report instrument ( Beck & Steer, 1993), center for Epidemiological Studies Depression Scale revised (CESD-R-10) ( Jackson *et al.*, 2022) and an online survey. This diversity of resources demonstrates, once again, the lack of consensus and the need to establish a more homogeneous protocol among researchers, in order to know what tools should be used in each aspect of mental health. Therefore, it would be necessary to lay the foundations to reach viable conclusions that can be extrapolated to swimmers to measure and diagnose mental health. All this evidence makes it necessary to carry out specific studies with swimmers and ex-swimmers, in order to carry out intervention protocols and prevention of mental health, rather than waiting for symptoms to appear during sports life or even waiting for the testimonies of retired swimmers.

In conclusion, mental health problems are a salient topic in sports since 1990s. Studies that have analysed mental health symptoms and disorders in competitive swimmers have been insufficient in the scientific literature. They have even valued it from different dimensions and tools, masking the problem or simply reporting data that have not helped to clarify what mental health is. Therefore, it is necessary to fill this gap with studies that work under the same consensus and in collaboration with specified technical teams, in order to supervise, evaluate and provide services, through detection tests and support to swimmers in their training, as well as before and after competitions. Moreover, there should be a direct involvement by the competent bodies and institutions to tackle, instead of ignoring or mismanaging this situation, in order to respond to the needs of swimmers. Finally, mental health is everyone’s responsibility, from the swimmer himself to the bodies and institutions involved, and interdisciplinary and collaborative work should be done, establishing specific programs based on the sport in order to guarantee efficient prevention and care.

### Preregistered data analysis

The author declares that he/she has not registered the study with an independent registry without a data analysis plan.

## Data Availability

All data underlying the results are available as part of the article and no additional source data are required.
