# Supplementary material for: Variables e instrumentos para evaluar la salud mental en nadadores de competición: una revisión narrativa
Source: F1000Res. 2024 Apr 18;12:1281. Originally published 2023 Oct 6. [Version 3] doi: 10.12688/f1000research.140504.3 (PMC11116940; doi:10.12688/f1000research.140504.3)
Supplement: Peer review history

# Reviewer report: Reviewer response for version 2

**DOI:** 10.5256/f1000research.159638.r228517

**Published:** 2024-05-23

**Authors:** Miguel Ángel Gómez Ruano

**Affiliations:** 1 Universidad Politécnica de Madrid, Madrid, Spain

The revision has improved the article and is suitable for approval.

Is the work clearly and accurately presented and does it cite the current literature?

Yes

If applicable, is the statistical analysis and its interpretation appropriate?

Not applicable

Are all the source data underlying the results available to ensure full reproducibility?

No source data required

Is the study design appropriate and is the work technically sound?

Yes

Are the conclusions drawn adequately supported by the results?

Yes

Are sufficient details of methods and analysis provided to allow replication by others?

Partly

Reviewer Expertise:

Sport Sciences

I confirm that I have read this submission and believe that I have an appropriate level of expertise to confirm that it is of an acceptable scientific standard.

# Reviewer report: Reviewer response for version 2

**DOI:** 10.5256/f1000research.159638.r229758

**Published:** 2024-03-21

**Authors:** Ricardo Zazo Sánchez Mateos

**Affiliations:** 1 University Miguel Hernández, Elche, Spain

The aspects to be assessed and taken into account, and which I recommend, are the following:

 a.     ABSTRACT

  

- Why are the keywords not in the language of the article (English)?

b.     DATA COLLECTION

  

- Why haven't you developed a bibliometric review in order to quantify the data?
- Why do you indicate that it is a narrative review, if in Figure 1. you include a flow chart? Key aspect of a systematic review.

Is the work clearly and accurately presented and does it cite the current literature?

Yes

If applicable, is the statistical analysis and its interpretation appropriate?

Yes

Are all the source data underlying the results available to ensure full reproducibility?

Yes

Is the study design appropriate and is the work technically sound?

Yes

Are the conclusions drawn adequately supported by the results?

Yes

Are sufficient details of methods and analysis provided to allow replication by others?

Yes

Reviewer Expertise:

sport science, psychology sports, swimming competition, prevention mental health

I confirm that I have read this submission and believe that I have an appropriate level of expertise to confirm that it is of an acceptable scientific standard.

# Response

**Published:** 2024-03-25

**Authors:** Alfonso Trinidad

**Affiliations:** Education and Humanities, European University- Campus Madrid, Madrid, Community of Madrid, Spain

a.     ABSTRACT

- Why are the keywords not in the language of the article (English)?

Dear reviewer, thank you for your comment. There was indeed an error when the keywords were uploaded to the platform. The error has been corrected and the keywords have been changed to English:

**Keywords**: Swimming, Competition, Sport, Wellness, Assessment, Monitoring, Detection, Prevention

 b.     DATA COLLECTION

- Why haven't you developed a bibliometric review in order to quantify the data?

Dear reviewer, in answer to your question, the aim of the study was not to carry out a quantitative analysis of scientific publications in order to identify and evaluate research trends and patterns within the field of mental health in sport in general. In our case, we focused our analysis on swimming and specifically on the instruments used and variables analysed within the scientific literature.

- Why do you indicate that it is a narrative review, if in Figure 1. you include a flow chart? Key aspect of a systematic review.

Dear reviewer, in response to your question, it has been decided to add a flowchart to the narrative review (Fig. 1) in order to give more emphasis and consistency to the research by graphically showing the steps or processes to be followed to reach the solution of the study problem.

# Reviewer report: Reviewer response for version 1

**DOI:** 10.5256/f1000research.153869.r213018

**Published:** 2023-11-16

**Authors:** Miguel Ángel Gómez Ruano

**Affiliations:** 1 Universidad Politécnica de Madrid, Madrid, Spain

The narrative review submitted is quite interesting, well-prepared, and useful for coaches and researchers interested in improving the mental health of their swimmers. I have some comments and suggestions in order to improve the meaning and readability of the manuscript.

  

- Please use the impersonal form when writing through the manuscript.
- Introduction, first paragraph, line 3, please write (WHO) after World Health Organization.
- Methods, authors should describe what type of review they are conducting, in this case, a narrative review. I suggest using the following reference to justify the methodology used: Grant, M. J., & Booth, A. (2009). A typology of reviews: an analysis of 14 review types and associated methodologies. *Health Information and Libraries Journal*, *26*, 91-108 ^1^.
- Method, please add the date when the search was made.
- Figure 1, should consider only 3 phases according to PRISMA 2020 guidelines, please modify it accordingly.
- Table 1 should be presented by pooling the studies that used the same methodology, then this would clarify the visual inspection of every study.
- The discussion could be enriched using an infographic that summarizes the main factors and variables under analysis.

Is the work clearly and accurately presented and does it cite the current literature?

Yes

If applicable, is the statistical analysis and its interpretation appropriate?

Not applicable

Are all the source data underlying the results available to ensure full reproducibility?

No source data required

Is the study design appropriate and is the work technically sound?

Yes

Are the conclusions drawn adequately supported by the results?

Yes

Are sufficient details of methods and analysis provided to allow replication by others?

Partly

Reviewer Expertise:

Sport Sciences

I confirm that I have read this submission and believe that I have an appropriate level of expertise to confirm that it is of an acceptable scientific standard, however I have significant reservations, as outlined above.

## References

[1] : A typology of reviews: an analysis of 14 review types and associated methodologies. *Health Info Libr J* .2009;26(2) : 10.1111/j.1471-1842.2009.00848.x 91-108 10.1111/j.1471-1842.2009.00848.x 19490148

# Response

**Published:** 2023-11-30

**Authors:** Alfonso Trinidad

**Affiliations:** Education and Humanities, European University- Campus Madrid, Madrid, Community of Madrid, Spain

Dear Reviewer,

 Thank you very much for your comments and suggestions. The changes have been made according to your instructions:

- Please use the impersonal form when writing through the manuscript.

The manuscript has been revised and the erroneous verb tenses have been changed to impersonal. Apologies for this.

- Introduction, first paragraph, line 3, please write (WHO) after World Health Organization.

Corrected. Thanks for the comment.

*Moreover, the World Health Organization (WHO) defines “global health” as “a state of complete physical, mental, and social well-being,”.*

- Methods, authors should describe what type of review they are conducting, in this case, a narrative review. I suggest using the following reference to justify the methodology used: Grant, M. J., & Booth, A. (2009). A typology of reviews: an analysis of 14 review types and associated methodologies. *Health Information and Libraries Journal*, *26*, 91-108 ^1^ .

The type of review used in the methodology was described. For this, the suggested reference was taken into account. Subsequently, the References section was updated.

*A narrative review or literature review was carried out where the materials published in the literature (tests and instruments to evaluate and diagnose mental health in highly competitive swimmers) were described, in order to perform a textual, tabular or graphic synthesis of Your results. and contributions (Grant & Booth, 2009).*

- Method, please add the date when the search was made.

Corrected. Thanks for the comment.

The start of the study was in February 2023 through combined search processes, in which potential studies, clearly planned and ordered, were identified. In orden to perform that, the following bibliographical databases were consulted: Dialnet, WOS (Web of Science) and Scopus with the following search terms included in Boolean search strategies: (mental*[tiab] AND (health [tiab] AND competitive [tiab] AND swimmers [tiab]) OR (swimming [tiab]) or in Spanish (mental*[tiab] AND (salud [tiab] AND competitivos [tiab] AND nadadores [tiab]) OR (natación [tiab]).

- Figure 1, should consider only 3 phases according to PRISMA 2020 guidelines, please modify it accordingly.

Corrected. Thanks for the comment.

- Table 1 should be presented by pooling the studies that used the same methodology, then this would clarify the visual inspection of every study.

Corrected. Thanks for the comment.

- The discussion could be enriched using an infographic that summarizes the main factors and variables under analysis.

Thanks for the suggestion. I will ask the magazine if this option is possible within the discussion section.
